# Metabolomic analysis of human vitreous humor differentiates ocular inflammatory disease

**Published:** 2009-06-13

**Authors:** Stephen P. Young, Maged Nessim, Francesco Falciani, Victor Trevino, Somnath P. Banerjee, Robert A.H. Scott, Philip I. Murray, Graham R. Wallace

**Affiliations:** 1 Department of Rheumatology, University of Birmingham, Birmingham, UK; 2 Academic Unit of Ophthalmology, University of Birmingham, Birmingham, UK; 3 Division of Immunity and Infection, University of Birmingham, Birmingham, UK

## Abstract

**Purpose:**

Vitreoretinal disorders lack specific biomarkers that define either disease type or response to treatment. We have used NMR-based metabolomic analysis of human vitreous humor to assess the applicability of this approach to the study of ocular disease.

**Methods:**

Vitreous samples from patients with a range of vitreoretinal disorders were subjected to high-resolution ^1^H-nuclear magnetic resonance spectroscopy (NMR). Good quality spectra were derived from the vitreous samples, and the profiles were analyzed by three different methods.

**Results:**

Principal component analysis (PCA) showed a wide dispersal of the different clinical conditions. Partial least squares discriminant analysis (PLS-DA) was used to define differences between lens-induced uveitis (LIU) and chronic uveitis (CU) and could distinguish between these conditions with a sensitivity of 78% and specificity of 85%. A genetic algorithm coupled with multivariate classification identified a small number of spectral components that showed clear discrimination between LIU and CU samples with sensitivity and specificity >90%. Assignment of specific resonances indicated that some metabolites involved in the arginase pathway were significantly more abundant in LIU than CU.

**Conclusion:**

The discrimination we observed based on PCA, PLS-DA, and multivariate variable selection analysis of the NMR spectra suggests that a complex mix of metabolites are present in vitreous fluid of different uveitic conditions as a result of the disease process. Collectively the data demonstrates the efficacy of metabolomic analysis to distinguish between ocular inflammatory diseases.

## Introduction

Vitreoretinal disorders encompass a complex group of diseases with different pathogenetic mechanisms including inflammatory and proliferative conditions. Many of these, such as uveitis, lack specific biomarkers that define either disease type or response to treatment. A range of possible endpoints is used to define outcome, but it is not clear how these relate to each other in different patients or studies [1]. This is particularly relevant for clinical trials when comparing a novel treatment to established therapy. Several studies have identified inflammatory mediators, such as cytokines, chemokines, and growth factors or single nucleotide polymorphisms with disease type, activity, and response to treatment, yet there is no clear result that can relate to clinical response [2-6]. This, in part, results from the narrowly focused analysis of particular candidate molecules or single nucleotide polymorphisms (SNP), selection of which is based on knowledge of disease type and the immune response. More recently, multiplex protein and gene analysis have been utilized in ocular disease and results have shown profiles of interest, although in the case of proteins, still within a selected group of molecules [7-9].

An even broader global profiling of the network of proteins or metabolites found in cells, tissues or fluids can increase our understanding of the multiple interacting processes involved in complex systems. One such approach is metabolomics, which analyses the metabolic consequences of gene expression and protein activity [10]. Metabolomics most often combines either high-field nuclear magnetic resonance or mass spectrometry of biofluids with pattern recognition (principal components analysis) within the resulting dataset. It is based on the concept that metabolic properties of tissues predispose to or are altered by disease processes, and these changes can be reflected in characteristic patterns (metabolomic profile) in blood, urine, or other body fluids.

Metabolomics has been applied to the study of coronary heart disease, where analysis of serum nuclear magnetic resonance (NMR) spectra appeared to distinguish between different degrees of coronary artery stenosis. The features in the NMR spectra contributing to this discrimination were largely lipid components and yet classical biochemical analysis of the usual lipid profiles was unable to discriminate between these patients [11]. Recently, metabolomic analysis has been reported to discriminate in the prognosis and diagnosis of other human diseases, including diabetes, blood pressure, and cancer [12-14]. In a study of prostate cancer a novel metabolic marker of potential prognostic importance was recently identified which was derived from a pathway not previously associated with this disease [15]. Thus it seems that the multiplexed analysis inherent in this approach, which takes into account all metabolite signals regardless of whether they have been specifically identified, is able to provide information not available by other means.

The analysis of eye disease using such approaches has been reported, but this has been limited to investigations of animal models. For example, significant changes in a number of metabolites in rabbit eye aqueous humor [16] and in rat lens [17] has been seen following UV-B exposure, and a rise in lactate in vitreous humor has been observed in a rabbit model of ocular hypertension [18]. High resolution analysis of human eye disease has not been reported but an analysis using in vivo NMR spectroscopy has identified lactate as a dominant metabolite in human vitreous [19] suggesting that high resolution metabolomic studies might reveal novel biomarkers in human ocular disease.

In this study we have analyzed metabolite fingerprints in vitreous humor from patients with various vitreoretinal conditions, using high-resolution ^1^H-nuclear magnetic resonance spectroscopy in conjunction with principal component analysis (PCA), partial least squares discriminant analysis (PLS-DA), and multivariate variable selection (MVS) for classification to determine if metabolic profiles could define disease states.

## Methods

### Patients

Patients were recruited from the tertiary referral Vitreoretinal Unit of the Birmingham and Midland Eye Centre. A total of 42 vitreous humor samples were obtained from patients undergoing pars plana vitrectomy for various vitreoretinal disorders: chronic non-infectious uveitis (CU; n=20), lens-induced uveitis LIU (n=9); proliferative diabetic retinopathy (PDR, n=2), proliferative vitreoretinopathy (PVR, n=2), rhegmatogenous retinal detachment (n=7), Candida endophthalmitis (n=1), and Varicella Zoster virus acute retinal necrosis (ARN, n=1). The CU patients comprised sixteen patients with panuveitis (two with sarcoidosis), two with intermediate uveitis (pars planitis), and two with Fuchs’ heterochromic cyclitis (FHC). All of the eyes in the CU group had chronic relapsing and remitting disease and were operated on at a time of remission. None had ‘burnt-out disease’, The indications for vitrectomy in the panuveitis and intermediate uveitis patients were vitreous opacities and cystoid macular edema with epiretinal membrane formation/vitreo-macular traction. None of these patients had any anterior chamber activity with the exception of FHC patients who had mild (+1) inflammation of the AC. Of these patients, all apart from 3 were receiving systemic immunosuppression. The indication for vitrectomy in the FHC patients was vitreous opacities, and none of these patients were on any topical or systemic corticosteroid therapy. The LIU group comprised patients that within the previous 14 days had undergone complicated phacoemulsification cataract surgery where the lens nucleus had inadvertently dropped into the vitreous cavity, resulting in an acute form of uveitis. All patients in the LIU group had active uveitis and were receiving topical corticosteroid, and none were on systemic anti-inflammatory or immunosuppressive therapy at the time of vitrectomy. Local Research Ethics approval and patient consent was obtained in all cases.

### Metabolomic analysis

An undiluted vitreous sample was taken at the beginning of surgery and was centrifuged at 300 rpm for 10 min and stored at –80 °C until analysis. After thawing, samples (0.1 or 0.2 ml) were centrifuged at high-speed (13,000x g), diluted 2:1 with D_2_O/H_2_O containing NaCl (150 mM), trimethylsilyl 2,2,3,3-tetradeuteropropionic acid (TMSP), and sodium phosphate (20 mM) pH 7.4. One-dimensional ^1^H spectra were acquired at 298 ^°^K using a standard spin-echo pulse sequence with water suppression using excitation sculpting on a Bruker DRX 500MHz NMR spectrometer equipped with a cryoprobe. Chemical shifts were calibrated with respect to the chemical shift position of the TMSP resonance. Spectra were segmented into 0.005 ppm (2.5 Hz) chemical shift ‘bins’ between 0.2 and 10.0 ppm using ProMetab version 2 and the spectral area within each bin was integrated [20]. Bins between 4.5 and 5.0 ppm containing residual water were removed.

The total spectral area of the remaining bins was normalized and the binned data describing each spectrum were then compiled into a matrix, with each row representing an individual sample. Finally, the columns were mean-centered before multivariate analysis.

### Statistical analyses

Principal components analysis (PCA) of the pre-processed spectra was conducted using PLS_Toolbox (Version 4.1; Eigenvector Research, Manson, WA) within MATLAB (version 7.5; The MathWorks, Cambridge, UK). PCA reduces the dimensionality of data and summarizes the similarities and differences between multiple NMR spectra using scores plots. This requires calculation of new variables (the PCs) that are weighted linear combinations of the original chemical shift bins (presented as loads plots).

Partial least square discriminant analysis (PLS-DA) was then employed using PLS_Toolbox. This validated [21,22] supervised analysis technique was used to build a training model to evaluate separation between groups based on known clinical diagnosis. The PLS-DA model was cross-validated using Venetian blinds [21,22],  a method which re-assigns randomly selected blocks of data to the PLS-DA model to determine the accuracy of the model in correctly assigning class membership.

Multivariate variable selection (MVS) for classification was performed using the GALGO package [23] on the pre-processed spectra. GALGO combines genetic algorithm driven multidimensional searches and statistical classification methods to find combinations of variables that can distinguish between sample classes. Our analysis identified 800 predictive gene subsets using GAs coupled to a k-nearest-neighbor classification method. These models were pooled and the 50 topmost frequent peaks were selected to design a single representative statistical model using a forward selection strategy. The best classification model was then chosen.

Provisional assignment of the major peaks in the spectra was done using Chenomx NMR Suite version 4 (Chenomx, Edmonton, Alberta, Canada) which is an NMR spectral analysis program for metabolic profiling, which provides library over 230 compounds which may be present in biofluid samples, in conjunction with the human metabolite database [24] and published literature [11,25]. Comparison of peak heights of selected metabolites was made using an unpaired Student T test.

## Results

**Figure 1:**
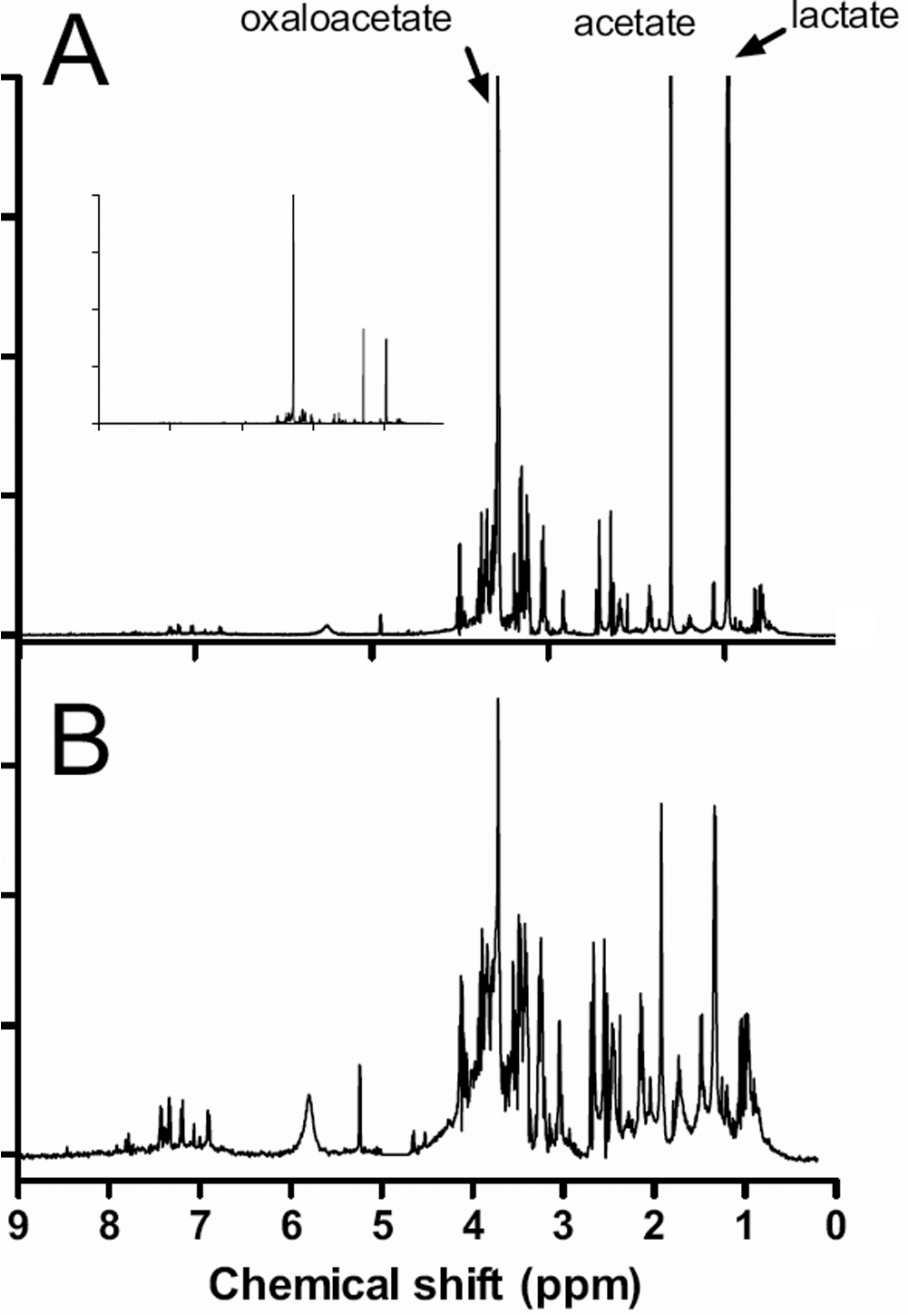
A typical 1D 1H NMR spectrum of vitreous eye fluid from a uveitis patient. **A**: The original spectrum labeled to show the regions in which some typical biofluid components are known to give rise to resonances. The spectrum has been scaled to highlight the detail; the inset is the unscaled whole spectrum. **B**: A data reduced spectrum in which the region between 4.5 and 5 ppm has been deleted so that any contribution from residual water signal is eliminated. This spectrum has been “binned” into 0.005 Hz regions.

NMR spectroscopy is a relatively insensitive method usually requiring samples to contain concentrations of target molecules in the high micromolar range. Our first concern was whether useful NMR spectra could be derived from the small vitreous humor samples available using a highly sensitive NMR instrument equipped with a cyroprobe that is able to minimize electronic noise in the system and thus maximize its sensitivity to the small NMR signals from dilute samples. Even with the small volumes available it was possible to acquire well-resolved spectra (Figure 1A). Acquisition time for each spectrum was relatively short, of the order of 10 min, making it possible to process a set of samples in a reasonable time. Although we used an NMR pulse sequence aimed at minimizing the detrimental effects of proteins present in the samples, which tend to produce amorphous humps within the spectra, this seemed to be less of a problem with the vitreous samples than with serum. The concentration of proteins in vitreous is considerably less than that in serum and this made a contribution to the quality of the spectra derived from these small volumes.

The NMR spectra are data rich entities but to make comparison and analysis possible some simplification is necessary. The first step is “binning” of the data to turn each spectrum from a continuous analogue signal into an array of data points. Each spectrum is segmented into approximately 2,000 regions and the area under each segment integrated. Since the concentration of individual samples can vary considerably it is necessary to normalize the total spectral area of each sample and the binned data describing each spectrum were then compiled into a matrix, with each row representing an individual sample. The spectra contained three dominant resonances (Figure 1A, inset) although many less-intense peaks in the spectrum were also well resolved (Figure 1A). To equalize the weightings of the smaller and larger peaks in the subsequent analysis every element was log-transformed (Figure 1B).

**Figure 2:**
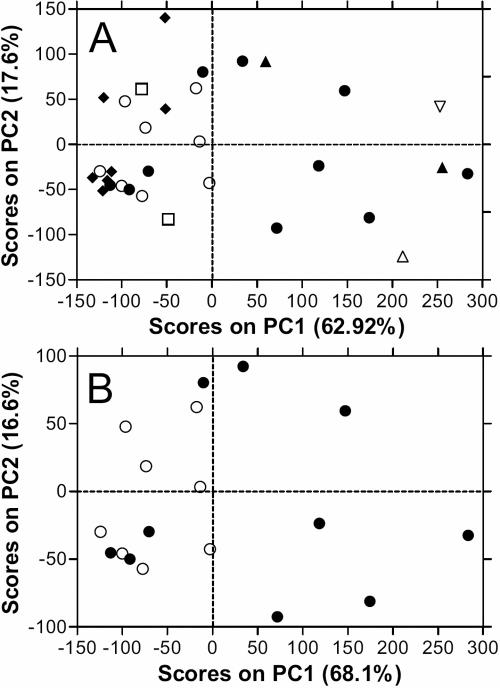
Principal components analysis of NMR spectra of vitreous fluid from uveitis patients. The processed and binned spectra were subjected to PCA analysis and the two major PC which accounted for 79.9% of the variance have been plotted. **A**: Shows all the samples from a broad range of uveitic conditions that were assessed: Chronic uveitis (●), Non-inflammatory – retinal detachment (◆), PVR (▲), PDR (□), ARN (△), Candida (∇), ERM (■) and Lens-induced uveitis (○). **B**: Vitreous from chronic uveitis and lens-induced uveitis patients Chronic uveitis (●), Lens-induced uveitis (○). This subset of patients was plotted since the PCA discriminated well with these conditions.

Principal Components Analysis (PCA) was then applied to the transformed datasets. With NMR data, PCA assesses the covariance around the mean of the data in each “bin” or region of the spectrum and assesses how these relate to each other. The principal components (PCs) extracted from the data describe the direction of the greatest variation in the data, and often a relatively small number of PCs (2 or 3) are able to describe the major covariance trends within the data. PCA analysis of the NMR spectra from 42 samples of vitreous fluid indicated that two principal components could account for over 75% of the covariance and these two PCs are plotted against each other (Figure 2A). Different disease groups showed a distinct separation on the plot of PC1 against PC2. Samples were distributed widely across the PC2 axis with no particular trends apparent. There is however discrimination based on the value of PC1, for example both PVR samples had positive PC1 values while both PDR samples had negative values. Similarly the patients with LIU were clustered with samples from patients with rhegmatogenous retinal detachment.

This discrimination was more apparent with the two largest groups of samples, LIU and CU. When these samples were assessed on their own, LIU samples were confined to the left side of the PC1 plot, suggesting a negative contribution of PC1 in these spectra while patients with CU were mainly confined to the right, suggesting a positive contribution of PC1 (Figure 2B).

**Figure 3:**
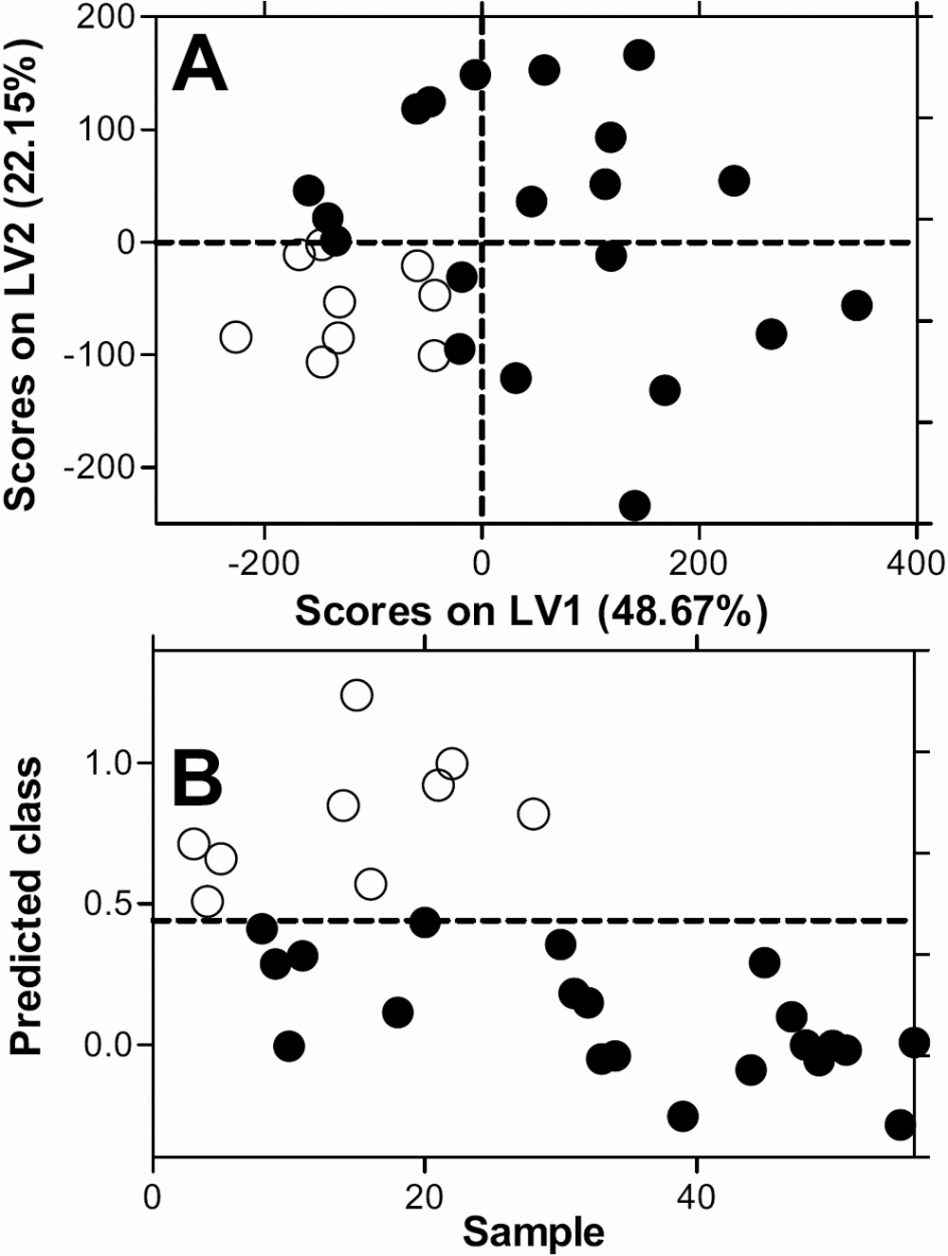
Partial least squares discriminant analysis (PLS-DA) of vitreous fluid NMR spectra from lens-induced uveitis and chronic uveitis patients. A PLS-DA model was constructed to predict diagnostic groups. **A**: Latent variable plot of the PLS-DA model, showing improved discrimination between LIU and chronic uveitis compared with the PCA analysis (Figure 2). **B**: Prediction of the group membership from the PLSDA. The model was cross validated, and was able to predict the classes, LIU and chronic uveitis, with sensitivity of 78% and specificity of 85%.

The supervised clustering analysis Partial Least Squares Discriminant Analysis (PLS-DA) was carried out to enhance the separation seen with the PCA. The PLS-DA model was cross-validated using Venetian blinds [21,22], a method which re-assigns randomly selected blocks of data to the PLS-DA model to determine the accuracy of the model in correctly assigning class membership. Using this approach an improved separation was seen between LIU and CU in the plot of Latent Variables (Figure 3A). PLS-DA allows a predictive model to be generated and this was able to accurately predict which class a sample came from (Figure 3B) with a sensitivity of 78% and specificity of 85%.

**Figure 4:**
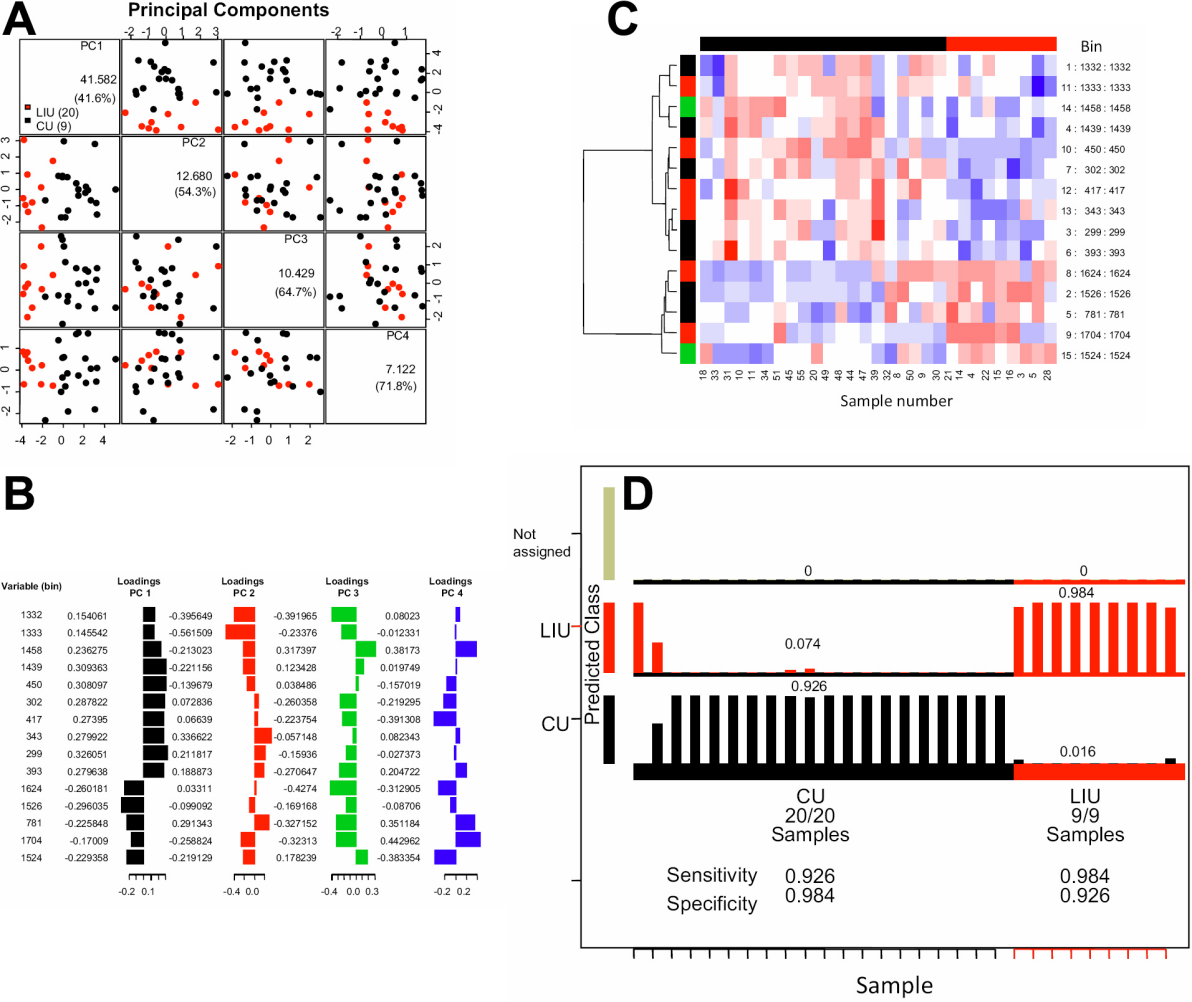
Multivariate variable selection of NMR spectra of vitreous from lens-induced uveitis and chronic uveitis patients. **A**: PCA analysis of LIU versus chronic uveitis following GA selection of NMR spectral bins which best discriminated between the conditions. 833 solutions were found in 2,199 search cycles matched with the corresponding spectra. **B**: Forward selection procedure using the 15 most frequent bins. The overall accuracy is shown in the vertical axis and the ranked bins in the horizontal axis. **C**: The best model containing the 15 most frequent bins generated by the forward selection procedure. Bins are drawn in the vertical axis whereas samples in the horizontal axis. Color means bin intensity. Hierarchical clustering from bins is shown for illustrative purposes. **D**: Prediction of class (disease) membership based on the best model. All samples were allocatable to one of the two classes and prediction of class membership could be made with sensitivity and specificity of over 90%.

To improve the classification further, a Genetic Algorithm (GA) approach was applied to remove variables that were not related to class differences. After GA several models were produced and in the best of these, the number of variables (NMR bins) was reduced to 13 that give a high discrimination power to classify the samples from patients with LIU and CU. The results showed that a selection of 13 bins from the spectra gave the maximum separation between the two sets of samples (Figure 4A), and the loadings of each bin used to define the different samples (Figure 4B). This model was confirmed by cluster analysis and Figure 4C shows a heat map displaying the relative contribution of each of these regions of the spectra which shows that the discrimination was dominated by a limited number of spectral regions, although many other areas across the spectra contribute to the power of the discrimination. This model was then used to classify each sample and all LIU samples were assigned to the correct predictive class (Figure 4D). Similarly, 18/20 of the CU samples were assigned to a unique group. The other two samples, although assigned to the CU group, were split between class I (CU) and class II (LIU) and these were the two samples from patients with FHC. The sensitivity and specificity for GALGO analysis was >90% (Figure 4D).

**Figure 5:**
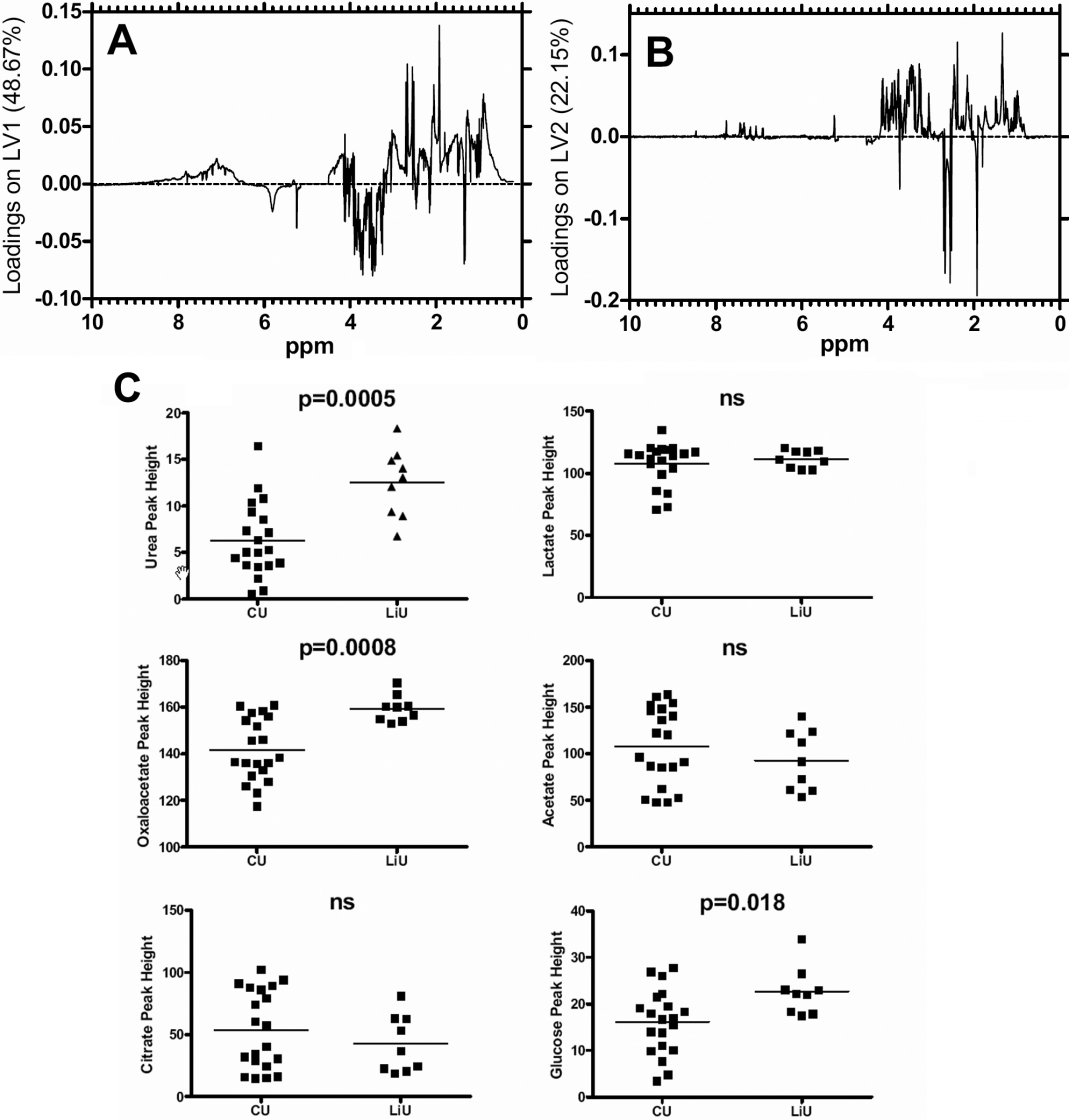
Assessment of the contribution of regions of the NMR spectra and specific metabolites to the discrimination between lens-induced and chronic uveitis. **A**: Loadings plots from the PLSDA analysis which indicates regions of the spectra which provide the greatest degree of discrimination between the two forms of uveitis. Positive peaks correspond to metabolites that are at higher concentration in the chronic relative to the LIU, and vice versa. **B**: Based on the original spectra and the loadings plot, peak heights of prominent metabolites resonances were plotted for each sample. Peak heights were compared using a T test.

All three analytical approaches indicated that the metabolic profile were sufficiently unique in LIU and CU to give a clear discrimination between these two groups. By plotting the contribution of each “bin” of the spectra to the covariance in the PLS-DA analysis it is possible to gain insights into categories of metabolites that may contribute to the apparent discrimination between samples. Such loadings for LV1 and LV2 for the data from LIU and CU in Figure 3, can be plotted. It can be seen that the regions of the spectrum at 5.8 ppm and from 3 to 4 ppm make a negative contribution to LV1 as do a number of peaks between 1 and 3 ppm, while other regions make a positive contribution. The situation is different with the LV2 plot with three prominent peaks apparent, but since LV2 does not segregate the diseases (Figure 3) it suggests these peaks do not contribute to the differences observed. (Figure 5A,B)

Based on these loadings plots we tentatively identified a number of metabolites represented in the spectra. Direct comparison of the peak heights of a number of these showed some significant differences between LIU and CU. There was a significant increase in oxaloacetate (p=0.0006), glucose (p=0.026), and urea (p=0.0003) in LIU samples compared to CU. By comparison lactate, acetate and citrate peaks showed no difference between the samples (Figure 5C).

## Discussion

This study shows for the first time that metabolomic analysis of vitreous humor samples can define different types of vitreoretinal disease. Specifically, two forms of uveitis, LIU and CU, could be segregated, with samples from patients with another form of chronic uveitis FHC showing a similar analysis to LIU samples. Moreover the FHC samples could be separated from LIU samples by classification analysis. In proliferative disease, proliferative vitreoretinopathy (PVR) and proliferative diabetic retinopathy (PDR), samples could be separated, while non-inflammatory retinal detachment samples clustered together. Finally, individual peaks could be identified and differences in such metabolites shown to be significantly different in the LIU and CU vitreous samples. This data, though preliminary, shows that metabolomics could potentially be useful in the diagnosis of, and identification of biomarkers in vitreoretinal disease. There are potential sources of bias in this study that relate to the patient population. Patients with CU, with the exception of individuals with FHC were on systemic treatment for their conditions, while LIU patients received topical steroids. However, although the extent of inflammation may differ between the groups, it only forms part of the metabolic profile and treatment alone does not directly influence the metabolites. Secondly, it is not possible to obtain longitudinal samples to clearly address the effect of treatment, however, current studies on serum and urine samples will clarify this issue.

Regardless of such caveats, the separation of LIU and FHC from CU samples is particularly interesting. LIU could be termed phacoantigenic and is an acute granulomatous reaction that probably represents an antigenic reaction against proteins released from the retained lens fragments after disruption of the lens capsule [26]. CU samples were predominantly from patients with idiopathic disease with the exception of one patient with sarcoidosis. Although there were only two FHC samples, this condition has a distinct clinical phenotype from other types of uveitis, and several previous studies have identified differences between FHC and CU patients in vitreous cellular infiltrate and cytokine production, suggesting a Th1 mediated response with CD8^+^ cells and few B cells as characteristic of FHC [27,28]. Recent studies have linked a positive antibody titer to rubella virus to FHC, suggesting a viral etiology supported by the cytotoxic T cell findings [29,30]. By comparison, idiopathic CU is by definition long-standing, mediated by T lymphocytes and inflammatory cytokines. How these different etiologies impact on the metabolome is a current area of interest.

The presence of lactate in human vitreous humor has been reported based on observations using in vivo NMR spectroscopy with a voxel focused largely on the vitreous region [19]. Variations in vitreous lactate levels following induced ocular hypertension in the rabbit have also been reported [18]. We can confirm here that lactate is present as a major metabolite in human vitreous, suggesting limited availability of oxygen within the eye. However, we saw no significant variation in the levels between chronic and lens induced uveitis, suggesting that the inflammatory processes were not inducing changes in oxygen demand or delivery. The significant differences in oxaloacetate and urea we observed are of interest in these inflammatory conditions as both molecules are involved in the urea cycle. In particular, urea is produced in the metabolism of arginine to ornithine, a process that has been described in both activated macrophages and endothelium at the expense of nitric oxide production [31]. In macrophages, alternatively activated cells (M2) produce ornithine in response to lipopolysaccharide (LPS), and ornithine stimulates cell proliferation while nitric oxide is known to inhibit such a response [32,33]. Similarly, ornithine synthesis has been described in human umbilical vein endothelial cells (HUVEC) stimulated with cytokines and LPS, which induce endothelial cell proliferation [34]. That levels of these molecules were significantly different in samples from patients with LIU may be due to the nature of the conditions, LIU presents as an acute response to lens fragments penetrating the vitreous cavity, while CU is by definition from patients with longstanding disease.

The cellular infiltrate present in uveitic vitreous suggests that leukocyte-derived metabolites may contribute to the profiles seen, since many of the metabolites appear to be derived from intracellular metabolic pathways. However, resident cells might produce other metabolites so the discrimination we have seen may better reflect the total cellular activity rather than metabolite production from just infiltrating cells. While we have attempted to assign peaks to known metabolites, the contribution of the whole spectrum of molecules may be critical in its usefulness in disease assessment and the MVS model we built confirms this and shows that as few as 13 regions of the spectra can be used to discriminate between samples. The different processes involved in these conditions that contribute to the metabolic profiles are supported by analysis of factors in vitreous fluid which showed growth factors and cytokines specific for each disease state [35].

In conclusion, this is the first description of metabolomic analysis of human inflammatory disease, and of vitreous samples in conjunction with appropriate bioinformatic analysis. The data shows a clear separation between different disease types with high sensitivity and specificity. While prospective validation studies on both metabolic profiles and metabolite identification are required, such global profiling is currently being tested with regards to prognosis, diagnosis and response to treatment in several disease states, and importantly whether such discrimination can be found in metabolic profiles from serum samples from these patients.
